# Social media and mobile applications in chronic disease prevention and management

**DOI:** 10.3389/fpsyg.2015.00567

**Published:** 2015-05-07

**Authors:** Eugenio Santoro, Gianluca Castelnuovo, Italo Zoppis, Giancarlo Mauri, Francesco Sicurello

**Affiliations:** ^1^Laboratory of Medical Informatics, Department of Epidemiology, IRCCS Istituto di Ricerche Farmacologiche Mario NegriMilano, Italy; ^2^Psychology Research Laboratory, Istituto Auxologico Italiano IRCCS, Ospedale San GiuseppeVerbania, Italy; ^3^Department of Psychology, Catholic University of MilanMilano, Italy; ^4^Department of Informatics, Systems and Communication, Università degli Studi di Milano-BicoccaMilano, Italy

**Keywords:** social media, mobile health, prevention, health promotion, social network, chronic disease

Social media, online social networks and apps for smartphones and tablets are changing the way we communicate. According to a recent Pew Research Center survey, 73% of Internet users among US adults engage in social networking to access, create, and share contents (Duggan and Smith, 2013). The number of smartphone users is growing worldwide [56% of American adults are currently smartphone owners (Smith, 2013)], and millions of applications (most of them related to social media or other communication tools) are available on the Google Play or iTunes store.

The increased prevalence of chronic (and non-communicable) diseases in high-income countries is today largely attributable to the convergence of an aging population with the persistence of several risk factors, including physical inactivity, use of tobacco and alcohol, high blood pressure and cholesterol, stress, depression, and overweight and obesity. Many of these risk factors can be mitigated by health interventions and education, and communication tools could support healthy lifestyle and behavior change.

In the past years there was an increasing interest in the use of digital technologies to support these changes because they contribute to enhance levels of surveillance over behaviors and have the potential to provide acceptable and cost-effective interventions by transferring treatment, rehabilitation and prevention of a condition to self-care in the community (Castelnuovo et al., 2010).

Social media and smartphone-based applications are now changing how people interact with the healthcare and public health systems (Santoro, 2013; Santoro and Quintaliani, 2013). The participatory, interactive nature of social media platforms allows for information to be generated and shared in a viral fashion, and provide new mechanisms to foster engagement and partnership with consumers, to change their behaviors and to fight against unhealthy lifestyles. More than 100,000 health related apps are also available in the market allowing people to record, track, and analyze vital signs and physical health data over time, to obtain feedback and general information about the disease they suffer from, and to receive alerts to remind them to take their medications or to measure their blood glucose levels.

Due to their possible implications in public health a growing number of scientists suggests to incorporate social media and mobile health in health promotion and healthcare programs (Burke-Garcia and Scally, 2014).

## Social media and social network technologies for health promotion and management

Social media can directly support disease management by creating online spaces where patients can interact with clinicians and share experiences with other patients (Coiera, 2013). Cancer patients use Twitter to discuss treatments and provide psychological support (Tsuya et al., 2014) and online engagement seems to correlate with lower levels of self reported stress and depression (Beaudoin and Tao, 2008).

Wellness programs frequently incorporate social media to create a sense of community, group people around shared goals, and offer social and emotional support. A trial reported that adding online community features to an Internet-mediated wellness and walking program improves adherence and did reduce participant attrition (Richardson et al., 2010). Moreover randomized controlled trials demonstrated that Facebook-based intervention approaches is potential for increasing physical activity in young adult cancer survivors (Valle et al., 2013) and that engagement with Twitter is related to weight loss (Turner-McGrievy and Tate, 2013). Sharing health data among members of an online community seems also to be correlated to a better management of the disease they suffer from. A study of an online community enabling type two diabetic patients reporting, charting and optional sharing of recent hemoglobin HbA1c values through a geographic display, showed that patient provided data were current with 83.1% of most recent HbA1c values reported obtained within the past 90 days (Weitzman et al., 2011).

## Health and medical apps for smartphone and tablets for health promotion and management

The worldwide prevalence of mobile phones makes them a powerful platform for providing individualized health care delivered at the patient's convenience.

Mobile health (specifically smartphone plus health apps) could benefit hospitalized individuals in two general ways: allowing them to more easily and reliably self-diagnose their acute symptoms, and enhancing monitoring, tracking, and communication of different biometric information (e.g., blood pressure, glucose levels, spirometry values, oxygen saturation) for individuals with chronic medical conditions, moreover enabling greater engagement and partnership in their care (Steinhubl et al., 2013).

A wide range of mobile technologies has been developed and continues to be devised to better treat individuals with other chronic conditions, including hypertension (Kumar et al., 2015), diabetes and pulmonary diseases such as asthma and chronic obstructive pulmonary disease (Steinhubl et al., 2013).

Several studies have been conducted in order to evaluate their reliability suggesting that the quality of information is not generally evidence-based or did not match any clinical or medical guideline (Chomutare et al., 2011; Kumar et al., 2015). Similar studies demonstrated a diagnostic inaccuracy of smartphone applications for melanoma detection (Wolf et al., 2013).

Some randomized controlled trials have been conducted demonstrating the efficacy of medical apps in managing chronic diseases. For example a trial showed that in adjunct to usual care, the use of a diabetes-related smartphone application combined with weekly text-message support from a health care professional can significantly improve glycemic control in adults with type 1 diabetes (Kirwan et al., 2013). Patients who attended cardiac rehabilitation and used a smartphone-based application had greater improvements in cardiovascular risk factors and were less likely to be readmitted to the hospital within 90 days of discharge (Widmer et al., 2014). Users of a mobile app to self-manage back low pain showed greater improvement compared to the control group in every comparison of the critical physical, behavioral, and worksite outcome measures at 4-month follow-up (Irvine et al., 2015).

Health promotion and disease prevention is another area were health apps for smartphone and tablets could be very promising. However, few preliminary studies, mainly in the area of nutrition and obesity, have been conducted evaluating their efficacy. Researchers found that a mobile health app which enable users to track intake calories and physical activities might be more effective than traditional interventions to help patients lose weight (Carter et al., 2013) and that the addition of a personal digital assistant and telephone coaching can enhance short-term weight loss in combination with an existing system of care (Spring et al., 2013).

However, some limits should be outlined in the development of medical apps for the management and prevention of diseases. The first is that healthcare agencies, health organizations and universities are not fully involved in this process. A recent survey showed that for only 2.8% of the apps for the self-management of hypertension a real involvement was documented (Kumar et al., 2015). The second is that few health related applications are validated by the Food and Drug Administration, including those which transform a smartphone in a medical device, even if recent guidelines in US have been issued to regulate them (FDA, 2013).

## Conclusions

The incorporation of social media tools and mobile health apps into chronic diseases management and prevention is expected to grow in practice and importance as more people communicate online. As our society becomes increasingly connected through wireless devices and accustomed to sharing private matters such as health with others online, new challenges and opportunities will arise to leverage this information in a safe, dynamic, and timely manner.

A wider involvement of national and international health and professionals organizations, the adoption of clinical guidelines or gold standards, an increasing practice to submit health apps to Food and Drug Administration for validation, and the conduction of high quality, adequately powered randomized controlled trials evaluating the effectiveness of social media, online social networks, and medical apps on clinical outcomes (currently limited in numbers and methodology) could enhance their reliability and adoption.

### Conflict of interest statement

The authors declare that the research was conducted in the absence of any commercial or financial relationships that could be construed as a potential conflict of interest.
